# Assessing the role of ketogenic dietary therapy in ring chromosome 20 syndrome: A patient‐led approach

**DOI:** 10.1002/epi4.12387

**Published:** 2020-03-11

**Authors:** Donald Gordon, Allison Watson, Archana Desurkar, Laura Cowley, Thomas F. Hiemstra

**Affiliations:** ^1^ Ring20 Research and Support UK CIO Brentwood UK; ^2^ Neurology Department Sheffield Children’s NHS Foundation Trust Sheffield UK; ^3^ Patient Led Research Hub Cambridge Clinical Trials Unit Cambridge University Hospitals NHS Foundation Trust Cambridge UK; ^4^ School of Clinical Medicine University of Cambridge Cambridge UK

**Keywords:** co‐production, KDT, ketogenic dietary therapy, refractory epilepsy, ring chromosome 20 syndrome r(20)

## Abstract

Ring chromosome 20 syndrome (r(20)) is an ultra‐rare disease characterized by drug‐refractory epilepsy, cognitive impairment, and behavioral problems. Nonpharmacological treatments alongside antiepileptic drugs early after diagnosis may help reduce seizure frequency and preserve cognition. Ketogenic dietary therapy (KDT) has benefitted children with complex, refractory epilepsies, but its efficacy in r(20) is unknown. We assessed clinical prescription, implementation, and patient experience of KDT in r(20) through online surveys and a workshop. Forty‐two patients, families, carers, and 23 healthcare professionals completed the surveys. While nearly all patients were familiar with KDT, only half had tried it. Significant improvement in seizure activity, cognition, and alertness was reported; side effects were typically mild but with one report of increased seizure frequency. A high rate of co‐morbidity, older age at presentation, behavioral problems, and cognitive impairment can make implementing KDT in r(20) challenging. In the UK, NHS KDT services are predominantly available to pediatric patients, with very limited adult access. A health economic analysis illustrating reduced acute care costs or improved quality of life may support more widespread KDT implementation. Growing evidence supports KDT as an effective and safe intervention, but further research is needed to understand the mechanisms of r(20) and its interaction with ketosis.

## 1 INTRODUCTION

Ring chromosome 20 syndrome (r(20)) is an ultra‐rare epilepsy with fewer than 150 cases reported worldwide^1^. Sporadic genetic mutations cause a ring formation with a breakpoint in the p13q12.33 region of chromosome 20 with varying cellular mosaicism.^2^ The majority of patients present with drug‐refractory focal impaired awareness seizures (previously called complex partial), nocturnal frontal lobe seizures, and prolonged spells of nonconvulsive status epilepticus.^2^ Cognitive impairment, regression, and behavioral problems are frequent and can have a considerable impact on activities of living for both patients and their carers.

Treatment of r(20) is challenging. The degree of cellular mosaicism is associated with age at seizure onset, extent of learning disability, and dysmorphism, but not associated with response to treatment.^3^ Despite the development of more than 20 antiepileptic drugs (AEDs) over the past 30 years, there has been no significant decrease in the percentage of epilepsy patients, regardless of diagnosis, with uncontrolled seizures.^4^ As prompt seizure control is likely to help preserve cognitive function, therapeutic options aside from AEDs should be considered as early as possible in the disease course.

The ketogenic diet is a high‐fat, carbohydrate‐restricted diet that results in the generation of ketones. Ketogenic dietary therapy (KDT) can reduce seizures and improve behavior in children with complex, refractory epilepsy. There is a large body of literature, including observational studies and randomized controlled trials, that suggest KDT can reduce seizures by over 50%.^5^, ^6^ Anecdotal reports suggest that the KDT may also be effective in r(20), although the likely mechanisms for this effect remain to be elucidated.

Clinical use of KDT is established in the UK: NICE guidelines recommend KDT for children who have not responded to appropriate AEDs, but they do not address KDT access or availability within the National Health Service (NHS).^7^ Its provision and perceptions among healthcare users and families have been previously described.^8^, ^9^, ^10^ Professional (Ketogenic Dietitians Research Network (KDRD), KetoPag) and patient support groups (Matthew's Friends, The Daisy Garland) provide information, resources, and support for KDT implementation.

In June 2018, Ring20 Research and Support UK approached the Patient Led Research Hub (PLRH) with a proposal to investigate the use and efficacy of KDT in r(20). We aimed to:

- Assess the current use of KDT in r(20) by UK and European healthcare professionals.
- Assess the personal KDT experience of r(20) patients, families, carers.
- Prioritize KDT research most important to r(20) patients, families, carers, and healthcare professionals.

## 2 METHODS

### 2.1 Literature search and survey development

We conducted a systematic review of existing literature to determine (a) the use of KDT in r(20), (b) the use of KDT in similar complex drug‐refractory epilepsies, and (c) previous questionnaire‐based assessments of the use of KDT in epilepsy. Results informed the development of two online surveys to assess KDT use and experience in r(20) for healthcare professionals (HCPs) and patients, families, and carers (PFCs), and to gather basic demographic, diagnostic, and clinical care information to benefit the current understanding of r(20). The surveys were qualitative in nature with descriptive comments encouraged; patient and expert collaborators assessed content, accuracy, and readability prior to distribution.

### 2.2 Survey distribution and analysis

Surveys were advertized through social media and epilepsy and KDT charities. The HCP survey was additionally cascaded through EpiCARE (European Reference Network for rare and complex epilepsies), KDRD, KetoPag, and UK hospitals advertising specialist support for complex epilepsies. To understand real or perceived challenges and barriers to KDT, responses were sought from anyone with experience in r(20) regardless of familiarity or use of KDT. Surveys were accessed via SurveyMonkey, open from March 11 to April 28 2019. Responses were collated and summarized.

### 2.3 Workshop

**Figure 1:**
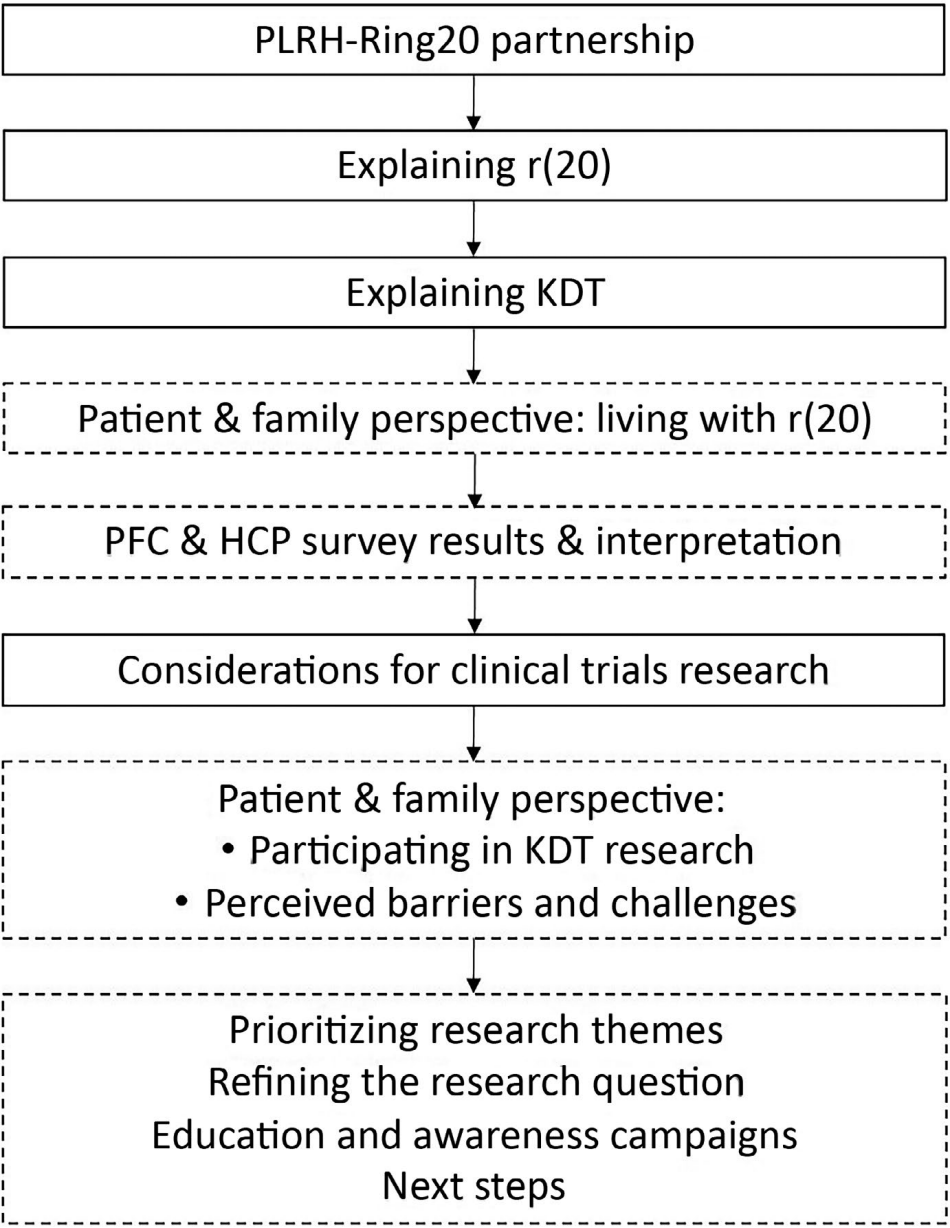
Workshop topics. Solid lines indicate medical or research expert speakers, dashed lines indicate patient speakers or group discussion

Expressions of interest for workshop attendance were sought through the Ring20 Charity patient and family, and healthcare professional networks. All interested members were welcome to attend; travel and accommodation costs were reimbursed. The workshop was hosted in London, May 2019; Figure 1 outlines the program. Attendees included Ring20 Charity co‐founders, two PLRH members, two r(20) patients, six family members and carers, two pediatric neurologists, pediatric dietitian specializing in KDT, and Mathew's Friends founder.

## 3 RESULTS

All survey data reported were supported anecdotally by workshop attendees.

### 3.1 Demographics

We considered the number of PFC responses to be significant (42 responses: 40 parents, 2 carers) given the ultra‐rare status of r(20): 17 responses were gathered from UK families where fewer than 30 are known to be affected. The HCP survey (23 responses: 14 neurologists, 6 dietitians or nutritional therapists, 2 pediatricians, 1 epilepsy specialist nurse) was initially intended for UK professionals but was expanded to Europe due to existing collaborations within complex epilepsies and ketogenic diet research.

Less than half (43%) of HCPs had experience treating at least one r(20) patient. 70% felt their experience was too limited to make a clinical inference regarding unique features of the condition. ADHD (14%) and autism (12%) were the most common co‐morbidities reported by PFCs. Seizure onset and r(20) diagnosis most often occurred by age 12 (93% and 74%, respectively); 48% of patients were adults. 81% of patients were prescribed more than 2 AEDs, with one PFC respondent indicating “6 or more.” Seizure activity was ranked the most important healthcare management aim by PFCs and ranked equally important as behavior and cognition by HCPs. The majority of patients had tried nondrug treatments (not including KDT), including vagal nerve stimulation (48%), medical marijuana (17%), steroids (14%), cognitive behavioral therapy (10%), food supplements, and other complementary medicine. PFCs indicated that quality of life would be most likely to improve through reduced AED side effects (76%), more effective medication (62%), and reduced pill burden (52%).

### 3.2 Ketogenic dietary therapy

All neurologist HCP respondents indicated willingness to prescribe KDT, with the majority (62%) reserving KDT for cases where 3‐4 AEDs have failed. 70% of PFCs recommended KDT by their clinician received advice more than 12 months after diagnosis. Nearly all (95%) of PFC respondents were familiar with KDT, although only 50% reported having tried it. KDT was mostly initiated from tertiary neurology services (PFC report: 45% outpatient, 40% inpatient, 85% with fasting) at 8‐12 (40%) or 13‐17 (40%) years old. Two patients (10%) had commenced KDT autonomously, with no professional support. 90% followed the diet as recommended for a minimum of 3 months, but only 20% maintained it for over 2 years. Nutritional supplements and specialist food products were recommended. Ketosis and r(20) symptoms were monitored through blood ketone levels (clinic 22%, home 40%) and seizure frequency (clinic 35%, home 45%). HCPs monitored safety by a combination of lipid profiles (35%), electrolytes (35%), serum liver and kidney tests (35%), and weight, height and head circumference (30%). 75% of PFCs noted a change in r(20) symptoms within 6 months of initiating the diet. KDT effect on r(20) symptoms and side effects are listed in Table 1. AED prescription remained largely unchanged while on KDT: reduced (20%) or withdrawn (10%) in a minority of cases, but neurologists indicated they would be willing to adapt prescription or dose as appropriate. Professional support was reported as “the perfect amount” (35%), “moderate” (30%), or “adequate” (20%) by PFCs, but this was less than support provision perceived by HCPs.

**Table 1:** Ketogenic diet therapy (KDT) effect on r(20) symptoms and side effects

PFC Survey: Did KDT affect r(20) symptoms? (n = 20)					
	Significant improvement	Small improvement	No change	Small worsening	Significant worsening
Seizure activity	30% (6)	15% (3)	40% (8)	10% (2)	5% (1)
Cognition and alertness	30% (6)	5% (1)	55% (11)	10% (2)	0% (0)
Behavior and mood	15% (3)	15% (3)	50% (10)	10% (2)	10% (2)
Sleep pattern	10% (2)	5% (1)	65% (13)	5% (1)	10% (2)
Free text: too early to infer (1 month KDT) (5%, 1); patient too unstable to infer (5%, 1)					

PFC Survey: Did you observe any side effects related to KDT? (n = 19)					
	Severe/ always present	Moderate/usually present	Mild/ frequently present	Very mild/occasionally present	Absent/never present
Gastrointestinal upset	11% (2)	16% (3)	21% (4)	37% (7)	16% (3)
Lost appetite	11% (2)	5% (1)	21% (4)	26% (5)	26% (5)
Lethargy	5% (1)	5% (1)	11% (2)	26% (5)	42% (8)
Bruising	5% (1)	5% (1)	0% (0)	0% (0)	74% (14)
Dehydration	0% (0)	0% (0)	5% (1)	47% (9)	32% (6)
Free text: patient too unstable to infer (5%, 1)					

HCP Survey: Did you observe any side effects related to KDT? (n = 8)					
	Severe/always present	Moderate/usually present	Mild/frequently present	Very mild/occasionally present	Absent/never present
Gastrointestinal	0% (0)	25% (2)	25% (2)	38% (3)	13% (1)
Linear growth	0% (0)	13% (1)	25% (2)	38% (3)	13% (1)
Dyslipidemia	0% (0)	0% (0)	50% (4)	38% (3)	13% (1)
Hypoglycemia	0% (0)	0% (0)	25% (2)	63% (5)	13% (1)
Nephrolithiasis	0% (0)	0% (0)	25% (2)	50% (4)	25% (2)
Dehydration	0% (0)	0% (0)	25% (2)	38% (3)	25% (2)
Bone density	0% (0)	0% (0)	13% (1)	63% (5)	13% (1)
Lethargy	0% (0)	0% (0)	13% (1)	63% (5)	13% (1)
Anorexia	0% (0)	0% (0)	13% (1)	63% (5)	13% (1)
Bruising	0% (0)	0% (0)	0% (0)	38% (3)	50% (4)
Pancreatitis	0% (0)	0% (0)	0% (0)	13% (1)	88% (7)
Cardiomyopathy	0% (0)	0% (0)	0% (0)	0% (0)	100% (8)
Free text: increased seizure frequency (13%, 1)					

Note

Figures displayed as percent of response relative to question (number of responses). HCP side effect question was completed by 5 neurologists, 1 dietitian, 1 pediatrician. PFC—patient, family, carer; HCP—healthcare professional.

### 3.3 Challenges and barriers

Patients, families, and carers reported maintaining the diet while socializing or dining out was the most challenging aspect of KDT (60% “extremely” or “very difficult”); restricted meal options (55%), food taste and consistency (50%), and planning ahead (50%) were also major challenges. Conversely, affording extra/specialist groceries and understanding recipes/nutritional guidelines were not perceived as troublesome (each 20% “extremely” or “very difficult”). Patients discontinued KDT because of perceived ineffectiveness (35%), poor taste/diet restrictiveness (30%), negative side effects (30%), and/or concerns over long‐term health impact. It was noted that the current provision of adult KDT services within the NHS is limited, leading to some patients withdrawing from the therapy and others unable to initiate it. Some hospitals were unable to cater to KDT needs required by pediatric inpatients. HCPs report barriers to KDT prescription as patient compliance concerns (39%), patients uninterested or concerned over side effects/impact (30%), and/or lack of funding (17%). PFC respondents that had not experienced KDT (50%) reported they did not want to try it because of poor taste/diet restrictiveness (33%), clinical advice (24%), difficulties presented by co‐morbidities (24%), and/or concerns over effectiveness, long‐term health impact, and limited time and resources for meal preparation.

### 3.4 Research priorities

Nearly one‐third (32%) of PFCs indicated they would be willing to follow a prescribed ketogenic diet as part of a research study; an additional 34% indicated “I’m not sure.” Potential research design and ethical considerations (eg, randomized controlled trial across KDT centers including cluster‐randomized designs) were further discussed at the workshop. Attendees also considered an ongoing trial assessing KDT in children under 2 years old (NCT02205931), an r(20) natural history and biomarker study in set‐up (University of Glasgow), and the existing opportunity to collect KDT safety and efficacy data specific to r(20) through wider EpiCARE studies. In conclusion, attendees strongly prioritized improving KDT access and awareness as new research. A health economic analysis presenting the complexities of r(20) care, health utilities, and lifelong impact on quality of life for patients and families could highlight funding gaps, contribute to NICE access guidance, and encourage nationally agreed tariffs for complex epilepsies and dietary interventions. Establishing adult KDT services was considered paramount. An education campaign driven by Ring20 and KDT charities to improve awareness of dietary therapies and currently available support may also improve uptake and adherence.

## 4 DISCUSSION

We present results of two comprehensive surveys consolidated by a workshop with r(20) patients, families and carers, and medical experts. To our knowledge, this is the first time KDT prescription and practice have been assessed in r(20). The majority of respondents ranked seizure control as the most important healthcare management aim. This, combined with the number of AEDs, complementary therapies, and nonprescription products used by patients, suggests the need for more effective frontline treatments. For some patients, KDT prompted significant improvement in seizure activity, cognition, and alertness. Side effects were most commonly mild with severe reactions absent or minimal. Nonetheless, close monitoring at home and in clinic is required, as one family and clinician reported an increase in seizure frequency.

The high rate of co‐morbidities, behavioral problems, and cognitive impairment in r(20) can make implementing KDT extremely difficult for some families. Adhering to KDT requires commitment and organization, and will very likely have a significant impact on all family members. KDT is felt to be particularly difficult to maintain in social situations, which is an important aspect to consider as older teenagers and adults with r(20) are keen to lead normal lives with appropriate levels of support. Patient support groups and forums, as well as on‐call professional support, can be essential to adherence.

Presently, NHS KDT is predominantly available for pediatric populations and many centers have extensive waiting lists. Despite the typical early age at seizure onset, r(20) diagnosis is generally delayed and KDT initiation may occur only in the later teenage years. Some patients continue KDT for years, and others repeatedly return to its variations as symptoms or personal circumstances change. As almost half of our patient survey respondents have already transitioned into adult neurological services, there is an obvious need for adult KDT programmes.

A growing evidence base supports KDT as an effective and safe intervention in many types of drug‐refractory epilepsies, but further research is needed to understand the mechanisms of r(20) and its potential interaction with ketosis. Although KDT may not be suitable for every r(20) patient, there is a strong consensus that it should be considered as an early intervention. Education campaigns to address patient and family concerns, and increase professional awareness may benefit KDT prescription, support, and adherence. Further refining and adherence to NICE guidelines may encourage early implementation.

R(20) patients and families are, by necessity, very engaged with their healthcare. The majority are willing to consider research participation, but it must be practical and realistic to fit within complex care routines. Collaborating with similar epilepsy groups could greatly benefit research recruitment and impact. In the first instance, a detailed health economic analysis illustrating reduced acute care costs and improved quality of life may encourage more widespread NHS KDT implementation.

## CONFLICTS OF INTEREST

None of the authors has any conflict of interest to disclose. We confirm that we have read the Journal's position on issues involved in ethical publication and affirm that this report is consistent with those guidelines.
